# 18-year-old with Abdominal Pain Due to Congenital Bowel Malrotation: A Case Report

**DOI:** 10.5811/cpcem.2021.11.52611

**Published:** 2022-01-28

**Authors:** Ellen McMahon, Michael Penfold, Meghan Cain

**Affiliations:** * Vanderbilt University Medical Center, Division of General Pediatrics, Department of Pediatrics, Nashville, Tennessee; † Mayo Clinic, Department of Pediatrics, Rochester, Minnesota; ‡ Mayo Clinic, Department of Emergency Medicine, Rochester, Minnesota

**Keywords:** case report, emergency medicine, abdominal pain, bowel malrotation, Ladd procedure

## Abstract

**Introduction:**

Congenital bowel malrotation resulting in midgut volvulus is traditionally regarded as a diagnosis of infancy. Rarely, congenital bowel malrotation is diagnosed in adolescents or adults and requires a high index of suspicion. Presentations can be acute or chronic, and physical examination findings are nonspecific. Diagnosis is primarily achieved through abdominal computed tomography (CT) or during exploratory laparotomy. The pathophysiology in late-onset malrotation is similar to neonatal malrotation, with a division of Ladd’s bands – peritoneal fibrous bands that connect the cecum to the right lower quadrant retroperitoneum – as the definitive treatment. We present a case of congenital bowel malrotation in an adolescent with persistent and worsening migratory abdominal pain.

**Case Report:**

An 18-year-old female presented to the emergency department with two days of poorly localized abdominal pain and nausea. Initial evaluation was unremarkable and she was discharged home with a diagnosis of constipation. She returned two days later with worsening abdominal pain and new onset emesis. Given her persistent and worsening symptoms an abdominal CT was performed, which revealed malrotation of the bowel. Taken together, her CT findings and abdominal symptoms were concerning for symptomatic congenital bowel malrotation and she underwent a Ladd procedure. She remained asymptomatic both at discharge and at two-week postoperative follow-up.

**Conclusion:**

Symptomatic congenital bowel malrotation is more common in older children and adults than has traditionally been thought. Physicians must consider this diagnosis in their differential when working up a patient for acute or chronic intermittent abdominal pain to prevent potentially severe sequelae.

## INTRODUCTION

An 18-year-old female presented to the emergency department (ED) with acute onset of diffuse abdominal pain and nausea without vomiting. Initial differential diagnosis included etiologies of abdominal pain that are commonly considered in this age group in the emergency setting, such as appendicitis, constipation, obstruction, inflammatory bowel disease, urinary tract infection, pregnancy, cholecystitis, ovarian cyst, and ovarian torsion. The ED evaluation, including ultrasound imaging of the appendix was reassuring, and she was discharged to home with treatment for constipation. She returned to the ED two days later with severe right lower quadrant (RLQ) abdominal pain and non-bloody, nonbilious vomiting. A thorough workup was notable for congenital bowel malrotation; symptoms were relieved following surgical treatment. This case demonstrates that, while traditionally thought of as a diagnosis of infancy, congenital bowel malrotation should be considered in the differential diagnosis of older children, adolescents, and adults with abdominal complaints. Surgical management is the definitive treatment and leads to resolution of abdominal symptoms.

## CASE REPORT

An 18-year-old girl with a history of migraine headaches, allergic rhinitis, ovarian cysts, and multiple food allergies was referred to the ED with complaints of poorly localized abdominal pain and nausea without vomiting. She had presented to her primary care physician (PCP) earlier in the day with similar complaints and was noted to have decreased bowel sounds and diffuse abdominal tenderness to palpation. At that time, her PCP recommended she proceed to the ED for further evaluation. She had been started on omeprazole one month prior for presumed gastroesophageal reflux disease and endorsed a longstanding history of constipation. In the ED, she complained of two days of intermittent, migratory, cramping abdominal pain associated with diarrhea. Vitals signs were within normal limits. Her exam was notable for tenderness in the epigastrium and RLQ. Gallbladder and appendix ultrasounds (US) were negative for cholelithiasis, cholecystitis, or appendicitis. The patient’s abdominal pain improved over a matter of hours, and she was discharged home with instructions to return to the ED if her symptoms returned.

The patient returned to the ED two days later with worsening abdominal pain. It was rated at a 10 of 10 in severity, stabbing in nature, located in the RLQ, with associated nausea and non-bloody, nonbilious vomiting. She was afebrile, tachycardic, and had flushing of the face, neck, and chest. She had an intrauterine device (IUD) and noted two days of bright red vaginal bleeding that she felt was different in quality than her typical menses. She reported having a bowel movement the previous day without blood in the stool, and her diarrhea had resolved. On exam, she had tenderness to palpation in the RLQ and right flank. Physical exam was otherwise unremarkable. Differential diagnosis at this time included gallbladder pathology, such as cholelithiasis or cholecystitis, or appendicitis not seen on initial US, inflammatory bowel disease, irritable bowel syndrome, pancreatitis, urinary tract infection, pyelonephritis, abdominal migraine, or pelvic pathology such as ovarian torsion, ovarian cyst, or ruptured ectopic pregnancy.

**CPC-EM Capsule**

What do we already know about this clinical entity?

*Congenital bowel malrotation resulting in midgut volvulus is typically regarded as a diagnosis of infancy and can result in bowel necrosis, a surgical emergency*.

What makes this presentation of disease reportable?

*Congenital bowel malrotation is rarely considered in the differential diagnosis of patients presenting with a chief complaint of abdominal pain*.

What is the major learning point?

*It is a rare but increasingly reported cause of abdominal pain in adolescents and adults. The presentation is highly variable and may include acute or chronic abdominal pain*.

How might this improve emergency medicine practice?

*Emergency clinicians should consider congenital bowel malrotation in the differential diagnosis, particularly for patients who present multiple times*.

Initial labs were obtained and included complete blood count, C-reactive protein, hepatic function panel, lipase, coronavirus disease 2019, and urine pregnancy test, all of which were negative or unremarkable. Laboratory studies were notable for a bicarbonate of 18 milliequivalents per liter (mEq/L) (reference range: 23–30 mEq/L) and an anion gap of 16 mEq/L (3–10 mEq/L). Transabdominal and transvaginal pelvic US were negative for ovarian torsion, cysts, or ectopic pregnancy. Her IUD was noted to be in the proper position. Given progression of symptoms and prior unremarkable abdominal US, an abdominal computed tomography (CT) with intravenous (IV) contrast was performed. Abdominal CT demonstrated congenital bowel malrotation with small bowel on the right side and colon on the left side of the abdomen (Image). The appendix was identified and normal in appearance. There was no evidence of bowel obstruction or active bowel inflammation on CT. All other identified organs, including ovaries, gallbladder, liver, spleen, and kidneys, were normal in appearance.

The patient was given IV fluids, morphine for pain control, and ondansetron for nausea. Pediatric surgery was consulted for consideration of surgical intervention. The patient was admitted to the hospital for pain control and brought to the operating room three days after her initial presentation for a laparoscopic Ladd’s procedure given her CT findings of bowel malrotation without alternative diagnosis. Her abdominal pain was thought to be secondary to intermittent volvulus. Intraoperatively, the gallbladder, uterus, and ovaries were normal in appearance. The appendix was grossly normal; however, an appendectomy was performed and sent for pathology. There were few adhesions noted between the right colon and the right abdominal wall. The duodenum had numerous adhesions between the liver and small bowel. She tolerated the procedure without any complications.

The patient’s symptoms of abdominal pain and nausea improved postoperatively, and she was discharged to home two days after the procedure. On post-hospital follow-up two weeks after discharge the patient reported complete resolution of gastrointestinal symptoms. Pathology demonstrated an appendix with minimal focal mucosal inflammation and without perforation or fecalith.

## DISCUSSION

Congenital bowel malrotation results from a disruption of normal embryologic development of the intestine when the midgut fails to rotate around the superior mesenteric vessels.^1^,^2^ The total incidence is thought to be approximately one in 500 to one in 6000 live births.^3^,^4^ It is most often thought of as a disease process of infancy, and it is estimated that between 64–80% of cases present within the first month of life and approximately 90% within the first year. Malrotation results in a bowel with a narrow base of mesenteric fixation, which is a risk factor for the development of midgut volvulus.^5^ Midgut volvulus is an emergent complication that occurs when the bowel twists around the superior mesenteric artery axis, which can result in intestinal necrosis.^6^ In infants, this presents most commonly with bilious emesis, prompting an emergent workup using upper gastrointestinal fluoroscopy and barium contrast to reveal malposition of the duodenal-jejunal junction.^7^ In older children and adults, presentation is more varied: symptoms may include recurrent episodes of colicky abdominal pain, nausea, vomiting, and diarrhea, or may have a more acute presentation.^8^,^9^,^10^ Chronic intermittent symptoms in these populations are likely due to intermittent volvulus or obstruction from Ladd’s bands.^11^

Malrotation of the bowel can be identified on abdominal CT with IV contrast by identification of the ascending colon on the left side of the abdomen with small bowel on the right.^5^ There is no reliable means to determine which patients with congenital bowel malrotation will develop complications, such as midgut volvulus, as some patients remain asymptomatic with the diagnosis only noted on autopsy.^3^,^8^,^10^,^11^

The definitive treatment for bowel malrotation is a Ladd’s procedure, in which the Ladd’s bands, the mesenteric bands extending from the colon across the duodenum, are divided. Associated adhesions are lysed to broaden the base of the mesentery, and in some cases a concomitant appendectomy is performed.^13^,^14^ This results in anatomical correction of the anatomy with the small bowel repositioned to the right side of the abdomen and the colon on the left.^13^

Some clinicians suggest that patients with chronic symptoms or asymptomatic patients with incidental discovery of congenital malrotation should undergo a Ladd’s procedure as there is no way to determine who may go on to develop future complications including midgut volvulus.^7^,^15^ It has been reported that up to 89% of patients with symptomatic congenital bowel malrotation will have complete resolution of symptoms following a Ladd’s procedure.^15^ Our case demonstrates a previously healthy adult with acute abdominal symptoms from congenital malrotation of the bowel that resolved after undergoing a Ladd’s procedure.

## CONCLUSION

While symptomatic congenital bowel malrotation has been traditionally thought of as a disease of infancy, this case illustrates that it must also be considered as a part of the differential diagnosis of abdominal pain in older children and adults. Given the lower degree of suspicion for this diagnosis in these populations, delays in diagnosis may result in increased morbidity as intestinal necrosis can result from volvulus secondary to bowel malrotation, and the time to surgical intervention is crucial in preventing this complication. This case further illustrates that uncommon etiologies for a common chief complaint must be considered when a patient presents on multiple occasions despite an unremarkable initial evaluation.

## Figures and Tables

**Image:**
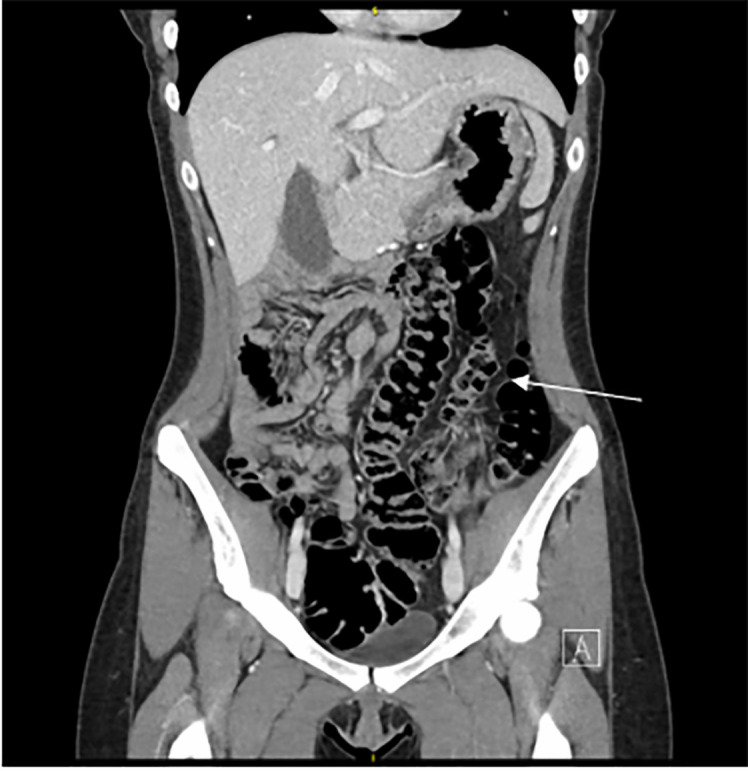
Abdominal computed tomography demonstrating congenital bowel malrotation with small bowel on the right side and colon on the left side of the abdomen. Arrow indicates the location of the colon on the left side of the abdomen.
